# Brain structural investigation and hippocampal tractography in medication overuse headache: a native space analysis

**DOI:** 10.1186/s12993-017-0124-5

**Published:** 2017-04-08

**Authors:** M. Meyer, G. Di Scala, M. Edde, B. Dilharreguy, F. Radat, M. Allard, S. Chanraud

**Affiliations:** 1 UMR 5287-CNRS, INCIA-Bordeaux University, 33076 Bordeaux, France; 2 Nuclear Medicine Department, Pellegrin Hospital, CHU Pellegrin, CHU of Bordeaux, Place Amélie Raba-Léon, 33000 Bordeaux Cedex, France; 3 Neurology Department, CHU Pellegrin, 33000 Bordeaux, France; 4 EPHE, PSL Research University, 75014 Paris, France

**Keywords:** Migraine, Pain, Macrostructure, Freesurfer, Hippocampus, Asymmetry

## Abstract

**Background:**

Spatial normalization of brain images, a prerequisite for voxel based morphometry analysis, may account for the large variability of the volumetric data in medication overuse headache (MOH); possibly because this disease concerns patients differing on both sex and age, and hence with different brain size and shape.

**Methods:**

The present study aimed at providing a subject-based analysis of macrostructure using a native space volumes segmentation (*Freesurfer*), and microstructure using a region of interest (ROI: i.e. hippocampus) tractography approach in MOH patients.

**Results:**

The results show that MOH patients had decreased volumes of left hemisphere temporal gyri (temporal superior, fusiform) and occipital middle gyrus, together with an increased volume of the left inferior (temporal) lateral ventricle. The left temporal volume was negatively correlated with depression score and medication dependence parameters. Seed-based tractography of the hippocampus revealed a decreased number of reconstructed fibers passing through the left hippocampus.

**Conclusion:**

To our knowledge, these alterations have not been described with methods involving brain normalization, and they indicate that left hemisphere temporal areas, including the hippocampus, may play a role in MOH pathophysiology.

*Trial registration number* NCT00833209. Registered 29 January 2009

## Background

Medication overuse headache (MOH) is a form of chronic headache [1] resulting from overuse of headache medications (simple analgesics, opioids and derivates, triptans). Neuroimaging techniques have provided important insight into the brain mechanisms supporting or, related to MOH; but discordance among published studies questions the significance and clinical relevance of these data. As a matter of fact, alterations of volumetric grey [2] and white matter [3] were described, but these findings have been challenged by other studies failing to find any structural alteration [4, 5]. The reasons for these discrepancies are still unknown, and can hardly be attributed to the methods used, as they were quite similar. In particular, most studies were based on whole brain exploration, such as voxel-based morphometry, a fully automated, hypothesis-free method, which provides a voxel-wise assessment of regional cerebral matter. Despite this intrinsic quality, these methods necessitate a normalization step. Spatial normalization is the conventional method for warping all subjects of a study into a common standard space. Although spatial normalization is intended to fit all brain images to a standardized space, the assumption that any voxel represents the same brain location for every subject is typically untrue. This may particularly be the case in structural neuroimaging studies of migraine, as they usually include patients differing on both age and gender, making brain structures difficult to coregister [6, 7]. Furthermore, although several studies have explored grey matter volumes, few reports are available for white matter, and to our knowledge, for cerebrospinal fluid, which can be an indirect marker of morphometric alterations. Here, we first aimed to specify whole-brain patterns of morphometric alterations in MOH patients. To this end, we investigated macrostructure of all brain tissues using a native space volumes segmentation approach. A secondary aim to the present study was related to previous findings obtained in the same sample of MOH patients who were compared to subjects with episodic migraine without medication overuse [4]; findings addressed the modifications of functional connectivity within one prominent resting-state network: the default-mode network (DMN). When compared to healthy controls, MOH patients had a decreased connectivity between the left precuneus seed and other regions of the DMN such as the frontal and parietal cortices, as well as an increased connectivity with the hippocampus and temporal areas. Both decreased and increased functional connectivity were related to duration of the disease, while increased functional connectivity in the hippocampus area was moreover related to medication dependence processes, as represented by the number of pills taken monthly [4]. In the context of these results, we made profit of the current native-space analysis to further investigate the hippocampal area and its connected neighbouring structures. We explored the hippocampal surrounding microstructure using a region of interest (ROI) tractography approach, and a fiber tract tool which enables modelling of fiber tract anatomy within each person’s own diffusion space. Here, we speculated that volumes and/or microstructure of specific brain regions, which would present morphometric alterations, would appear to be related to variables describing pathology processes.

## Methods

### Participants

MOH patients were recruited from the headache clinic of the Neurological Department of the University Hospital in Bordeaux. They were included if they fulfilled the diagnostic criteria for MOH with prior migraine (ICHD-II 8.2). The exclusion criteria were the following: post-traumatic headaches (ICHD-II 5.1 and 5.2); illness interfering with central nervous system functioning; psychotic disorder or current mood disorder; regular use of a psychotropic medication (antidepressant, benzodiazepine, antipsychotic drugs, but anticonvulsant medications were accepted). Healthy controls (HCs) were recruited among hospital staff members. The two groups were matched to have comparable age and sex distributions. We obtained local ethics committee approval and written informed consent from all subjects before study initiation.

### Disease characteristics

MOH patients had to provide information about the age at onset of migraine, the duration of migraine illness, the number of days with headache per month, the type of acute headache medication taken and the mean number of headache medication taken per month.

### Neuropsychological evaluation

All subjects filled in several self-administered questionnaires and scales: the Beck Depression Inventory (BDI-13 items [8]), the Pain Catastrophizing Scale (PCS [9]), the State Anxiety Inventory (STAI-state [10]), the Barrat Impulsivity Scale (BIS-11 [11]), and the Medication Dependence Questionnaire for Headache sufferers (MDQ-H [12]). They also underwent an Iowa Gambling Task (IGT [13]) in order to detect putative decision-making impairments.

### Magnetic resonance imaging (MRI) acquisitions

Patients were off medication for at least 12 h before the MRI acquisition. MRI scans were obtained using an ACHIEVA 3T scanner (Philips Medical System, Netherlands) with a SENSE 8-channel head coil. Anatomical high resolution MRI volumes were acquired in transverse plan for each subject using a 3D MPRAGE weighted-T1 sequence with the following parameters: TR = 8.2 ms, TE = 3.5 ms, 7-degree flip angle, FOV 256 × 256 mm^2^ to cover the whole brain, yielding 180 slices, no gap, voxel size 1 × 1 × 1 mm^3^. Two diffusion-weighted images with opposite polarity, allowing elimination of diffusion imaging gradient cross-terms, were performed using a spin echo single shot EPI sequence with the following parameters: TR = 7646 ms, TE = 60 ms, 90-degree flip angle, FOV 224 × 224 mm^2^, yielding 60 slices, no gap, voxel size 2 × 2 × 2 mm^3^. One b0 image was acquired and diffusion gradients were applied in 21 non-collinear directions (*b* value = 1000 s/mm^2^). To increase signal-to-noise ratio, the sequence was repeated in two successive runs for each polarity. All acquisitions were aligned on the anterior commissure–posterior commissure plan (AC–PC). For qualitative clinical readings, fluid-attenuated inversion recovery (FLAIR) images were also obtained with the following parameters: TR = 11,000 ms, TE = 140 ms, TI = 2800 ms, FOV 230 × 172 mm^2^, yielding 24 slices, gap of 1 mm, voxel size 0.72 × 1.20 × 5 mm^3^. The total scan duration, which also included resting state functional acquisition, was about 45 min.

### Volumetric analyses

FreeSurfer 4.5.0 (http://surfer.nmr.mgh.harvard.edu/) is a set of tools for automated cortical and subcortical reconstruction, volumetric segmentation and analysis of images; it parcellates the cortex into gross anatomical regions and produces statistics on volume for each region [14]. Briefly, this processing includes motion correction and averaging of multiple volumetric T1 weighted images, removal of non-brain tissue using a hybrid surface deformation procedure, automated Talairach transformation, segmentation of the subcortical white matter and deep grey matter volumetric structures (including hippocampus, amygdala, caudate, putamen), intensity normalization, tessellation of the grey matter and white matter boundary, automated topology correction, and surface deformation following intensity gradients to optimally place the grey/white and grey/cerebrospinal fluid borders at the location where the greatest shift in intensity defines the transition to the other tissue class. All stages of the stream are fully described by Fischl et al. [14].

To extract reliable volume estimates, Freesurfer’s semi-automatic anatomical processing was executed on the T1-weighted images of the two groups of subjects. The volume measures were therefore subject-specific. Grey matter was segmented into 148 regions, based on the Destrieux atlas [15]. Subcortical structures and ventricular system were segmented into 40 regions, based upon the existence of an atlas containing probabilistic information on the location of structures [16]. Finally, visual verifications were performed on every subject. In order to correct for inter-subject variability, each volume of the segmented regions was divided by the total intracranial volume for each subject.

### Tractography seeds

**Fig. 1:**
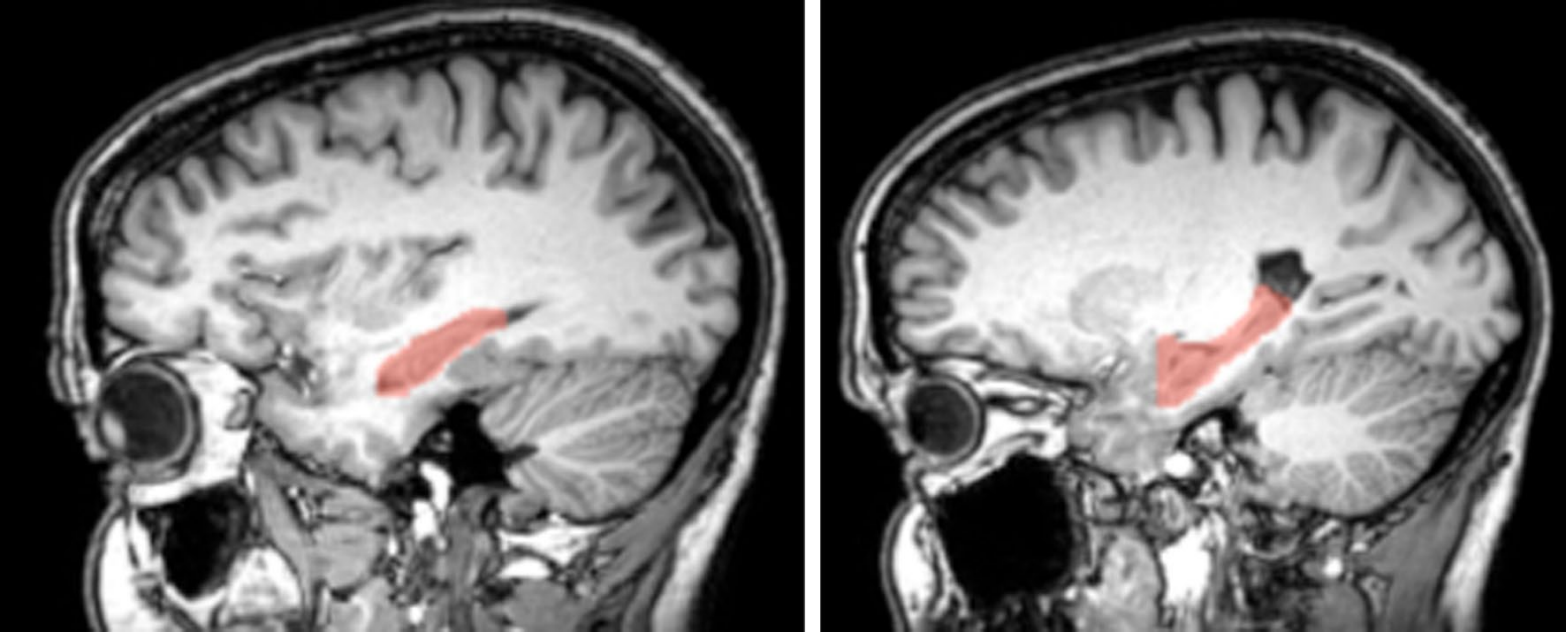
Example (from a control subject) of hippocampi masks as delimited on T2 scans and realigned on T1

The regions of interest (ROIs) were manually defined (co-author M.E.) using two atlases: the Atlas of Human brain, 3rd edition, 2007 and the atlas of Duvernoy [17]. We chose manual delineation and not FreeSurfer segmented label because previous study has shown that boundaries of the Freesurfer parcellation does not perfectly match with anatomical boundaries of this region specifically [18]. As seed masks for tractography analysis, the ROIs included the bilateral hippocampus, drawn on individual T2 scans (b0 diffusion unweighted image) (see Fig. 1 for an illustrative example of the seed mask).

The hippocampi were traced in the coronal plane with correction in the axial and sagittal planes. First, ROIs were delineated in the anterior part of the hippocampus (head) i.e., the posterior part of the amygdala to the tip of the tail of the hippocampus at the atrium. The temporal horn of the lateral ventricle (superior and lateral), the uncus of the apex then the Fimbria delimited the hippocampus body. A thin band of white matter was included along the Ammon Horn (inferior) and reached the Subiculum, which was included in its proximal part (median). Finally, the Crus Fornicis (superior), the lateral ventricle (lateral) and the Atrium defined the tail of the hippocampus.

Then, corrections in axial and sagittal planes were made on areas of white matter appearing in coronal plane. Finally, delimitation of the amygdala and boundaries with the pons were done on same planes. Regions of interest were enlarged with a two-voxel extension (4 mm) laterally to include white matter to ease tractography.

### Tractography

The fiber components of bundles emerging from ROIs were reconstructed using a voxel-by-voxel regularized streamline algorithm [19], which resembles diffusion tensor deflection [20, 21] and which is implemented in BrainVISA software (http://brainvisa.info/). This strategy, similar to diffusion tensor deflection, overcomes simple crossing configurations. At each voxel, two parameters were used to get the direction of the resulting tract: the tensor biggest eigenvectors directions with a weight of *α* (*α* representing local anisotropy) and inertia (that is, the incident direction of the tract) with a weight of 1 − *α*. The algorithm stepped forwards a distance of half the voxel size along this direction and did the computation again. The propagation mask excluded voxels likely to belong to grey matter (Fractional anisotropy FA < 0.2), and algorithm stopped for a maximum curvature angle adjusted for each bundle to 45° or when the tract length exceeds 200 mm. In addition to the number of reconstructed fibers, mean FA and mean ADC (Apparent Diffusion Coefficient) along the reconstructed fiber tracts were calculated.

In this article, the term ‘tracked fibers’ refers to the number of fibers generated by the algorithm in fiber bundles connecting predefined brain regions, rather than the actual number of fibers in anatomical fiber bundles (for further explanation, see [22]).

In order to correct for inter-subject hippocampal volume variability, the number of “fibers tracked” was divided by the hippocampal volume.

### Statistical analyses

Data were analysed using the Statistical Package for Social Sciences IBM SPSS 20.0. (IBM Corp., Armonk, NY). The significance level was set at p < 0.05. We studied variables distribution with the Kolmogorov–Smirnov test and brain regional volumes differing between groups using *t* tests for independent samples; no correction for multiple comparisons was performed.

Within the group of MOH, we calculated Spearman’s nonparametric correlations between brain regional volumes which differed between groups, disease characteristics and neuropsychological evaluation.

We calculated group differences for the ROI volume, and with respect to tractography results, that is number of reconstructed fibers as well as mean FA and mean ADC along the respective reconstructed fiber tract. Model check was performed using residual QQ plots and detection of outliers using leaf plots. If outliers were detected, they were removed from analyses.

## Results

### Population

Thirty-three subjects were included (sixteen MOH patients and seventeen HCs). One HC and one MOH patient were discarded due to invalid MRI data acquisition. Data from one MOH patient data were not entered in the hippocampal-related analyses because the tractography process could not launch. Data from one HC were not entered in analyses because reconstructed tractography variables were considered as outliers. Demographic and psychological characteristics of 14 MOH (10 women and 4 men) and 15 HC (12 women and 3 men) participants are presented in Table 1. Four MOH patients were overusing either opiates or a combination of analgesics containing opiates derivate. The total amount of acute headache medication taken per month was 60 [16–163] in the MOH group. Concerning neuropsychological evaluation, mean scores were significantly higher in MOH than HC for dependence (MDQ-H), depression (BDI), catastrophizing (PCS) and for anxiety (STAI). There were no between group difference for impulsivity (BIS) and decision making performance (IGT).

**Table 1:** Socio-demographic and psychological characteristics of subjects

Variables	MOH patients (n = 14)	Healthy controls (n = 15)	Significance (p)
Subjects’ characteristics			
Age (years)	45.1 ± 11.2	47.5 ± 10.1	0.550
Sex (F/H)	10/4	12/3	0.682
Migraine’s characteristics			
Migraine duration (years)	28.5 ± 11.9	NA	–
Age of onset of migraine	16.6 ± 9.3	NA	–
Medication Intake (per month)	59.8 ± 49.0	NA	–
Opiates abuse (yes/no)	4/10	NA	–
Caffeine abuse (yes/no)	3/11	NA	–
Prior withdrawal	3/11	NA	–
Neuropsychological evaluation			
MDQ-H score	87.7 ± 25.5	21.8 ± 2.1	<0.001*
BDI score	5.4 ± 4.5	1.1 ± 1.4	0.002*
STAI score	40.2 ± 11.2	29.8 ± 8.9	0.010*
PCS score	27.8 ± 12.6	5.1 ± 6.3	<0.001*
BIS score	57.1 ± 7.9	59.4 ± 7.1	0.411
IGT score	25.0 ± 26.3	16.0 ± 29.8	0.397

*BDI* Beck Depression Inventory, *PCS* Pain Catastrophizing Scale, *STAI* State Anxiety Inventory, *BIS* Barrat Impulsivity Scale, *MDQ-H* Medication Dependence Questionnaire for Headache sufferers, *IGT* Iowa Gambling Task, *NA* not applicable

* Significant between-groups difference at p < 0.001

### Regional volumes

Between-groups comparison of regional volumes revealed differences in both hemispheres (see Table 2), but patterns of morphological differences were different according to the brain side. In the left hemisphere of MOH patients, we observed an increase of the inferior lateral ventricle volume, and a decrease of the temporal superior, the fusiform and the occipital middle gyri volumes. We also found an increase of the frontal middle sulcus. In the right hemisphere of MOH patients, we observed an increase of the cingulum marginalis sulcus volume and a decrease of the choroid plexus volume.

**Table 2:** Brain regions showing between-group difference in volume

Regions	MOH patients (n = 14)	Healthy controls (n = 15)	Significance (p)
Left side			
Infero-lateral ventricle	0.00038 ± 0.00017	0.00025 ± 0.00012	0.027
Temporal superior gyrus	0.00112 ± 0.00022	0.00129 ± 0.00021	0.049
Fusiform gyrus	0.00356 ± 0.00066	0.00403 ± 0.00058	0.049
Occipital middle gyrus	0.00369 ± 0.00075	0.00428 ± 0.00078	0.048
Frontal middle sulcus	0.00236 ± 0.00057	0.00192 ± 0.00029	0.015
Right side			
Choroid plexus	0.00119 ± 0.00014	0.00135 ± 0.00022	0.025
Cingular marginalis sulcus	0.00186 ± 0.00041	0.00158 ± 0.00014	0.021

### Hippocampal tractography

**Fig. 2:**
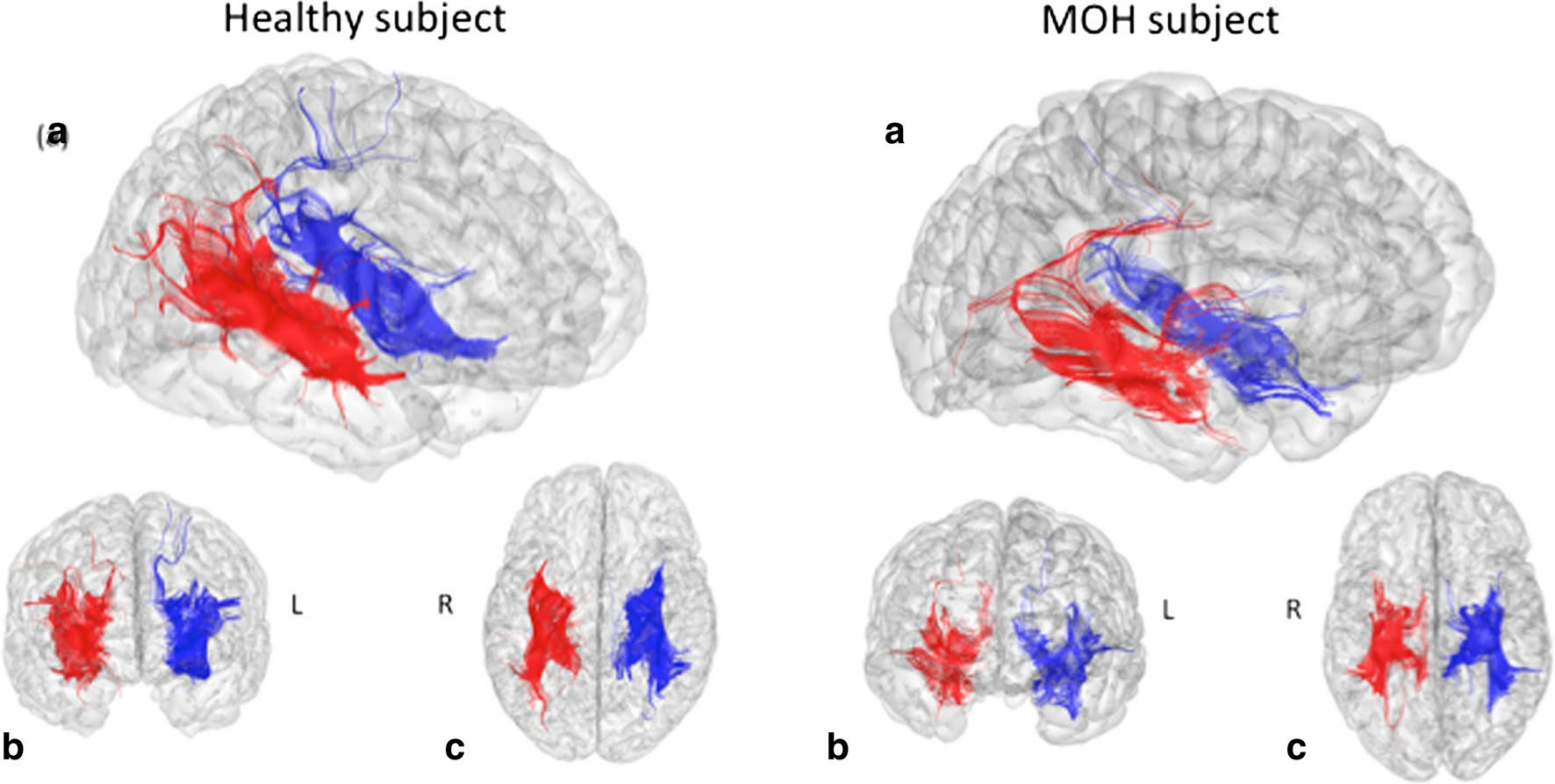
Tractography of the hippocampus fibers in one healthy subject (*left*) and MOH subject (*right*). Results are overlaid onto 3d view in **a** oblique plane, **b** frontal plane and **c** axial plane. Right side (*R*) in *red* and left side (*L*) in *blue*

Compared to healthy subjects, MOH patients had, on average, 29% fewer reconstructed fibers from the left hippocampus (see Table 3). No significant between-groups difference was found for the right hippocampus tractography. Neither FA nor ADC was modified within the two areas. A typical example of the tractography output in control subject and in MOH patient is shown in Fig. 2.

**Table 3:** Virtual number of reconstructed fibers in MOH patients and in controls, from the hippocampus separately in both hemispheres

	MOH patients (n = 14)	Healthy controls (n = 15)	Significance (p)
Right hippocampus			
Numbers of tracked fibers	536 ± 297	589 ± 263	0.605
Mean FA	0.65 ± 0.02	0.64 ± 0.03	0.746
Mean ADC (×10^10^ m^2^/s)	5.00 ± 0.21	4.98 ± 0.15	0.794
Left hippocampus			
Numbers of tracked fibers	473 ± 239	660 ± 269	0.045*
Mean FA	0.64 ± 0.03	0.64 ± 0.03	0.795
Mean ADC (×10^10^ m^2^/s)	5.01 ± 0.18	5.10 ± 0.15	0.177

Values of mean FA and ADC along reconstructed fibers

*ADC* apparent diffusion coefficient, *FA* fractional anisotropy

* Significant between-groups difference at p < 0.05

It is noteworthy that only in control subjects, the number of fibers in the left hemisphere was correlated with the number of fibers reconstructed in the right hemisphere, reflecting a brain asymmetry in MOH patients.

### Relationships between clinical data and MRI data

**Fig. 3:**
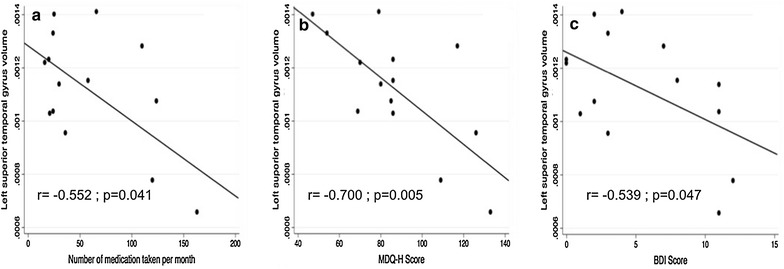
Significant relationships between volumetric data and either disease characteristics or neuropsychological data. **a** Relationship between the left temporal superior gyrus volume and the number of medication taken per month. **b** Relationship between the left temporal superior gyrus volume and the MDQ-H score. **c** Relationship between the left temporal superior gyrus volume and the BDI score

The volume of the left temporal superior gyrus was negatively correlated with the number of medication taken per month (r = −0.552; p = 0.041), the MDQ-H score (r = −0.700; p = 0.005) and the BDI score (r = −0.539; p = 0.047) (Fig. 3).

## Discussion

The present native-space study shows that MOH patients had decreased volumes of left hemisphere temporal superior gyrus, fusiform gyrus and occipital middle gyrus, together with an increased volume of the left frontal sulcus and the left inferior lateral ventricle. Left temporal volume was negatively correlated with depression score and dependence parameters (MDQ-H score and number of medication taken per month). Moreover, seed based tractography of the hippocampus revealed a decreased number of reconstructed fibers passing through the left hippocampus in MOH patients compared to HC. All of these findings, revealed in different types of analysis, highlight a brain asymmetry in MOH patients. These data suggest that left hemisphere temporal areas may play a specific role in pathophysiology of MOH.

### Grey matter/physiopathology

Cortical thickness is a marker of grey matter integrity, reflective of the size, density and arrangements of cells [23]. It is not static, changing over time in association with aging, brain usage patterns, and presence of brain diseases [24–26]. Previous studies have already shown that abnormal cortical thickness is present in both neurodegenerative disorders, such as Alzheimer’s disease, and acute or chronic pain disorders such as migraine [27–29]. Interestingly, the pattern of modifications obtained was reminiscent of what is found in anxiety [30], depression [31], or dependence [32]. In accordance to the pattern of relationships between brain and clinical variables established in the present study, multiple psychological states may contribute to the overuse of acute medications, including fear of headache, anticipatory anxiety, obsessional drug-taking behaviors, depression and, psychological drug dependence.

Another potentially important result is the enlargement of the left infero-lateral ventricle in MOH. It is well known that ventricular enlargement reflects changes in both the white matter and grey matter of the brain. Previous studies have shown that brain atrophy is accompanied by ventricular enlargement in normal aging and in neurodegenerative disease [33, 34]. Several studies have researched brain alterations in migraine, but few have focused on MOH patients, and to our knowledge, none of them have investigated cerebrospinal fluid system. Here, ventricular volume was related to temporal grey matter volume which was itself negatively correlated with depression score test, and dependence parameters, such as MDQ-H test and number of medication taken per month. All of these results add evidence for dependence processes being part of the pathophysiological mechanisms involved in MOH disease.

### Hippocampal tractography

The secondary aim of this study was to characterize hippocampal area’s microstructure in MOH patients. In our previous study [4], we reported decreased functional connectivity between precuneus and areas of the “Default Mode Network”, with increased functional connectivity between precuneus and the hippocampal/parahippocampal area. Using a deterministic algorithm, tractography of the hippocampus revealed a decreased number of fibers in left hippocampus, without change of any diffusion parameters. One limitation in our study is that we did not use any other brain region for selecting/separating the fibers tracked. In consequence, it is not possible to identify with confidence which brain anatomical bundles would be specifically concerned here. However, these results were left-sided whereas the previous ones concerning higher functional connectivity between the precuneus and the hippocampus were right-sided. To us, the most evident explanation would be that increased functional connectivity may be secondary to the anatomical alterations present in the left hemisphere.

### Advantages of native space analysis

Several studies have researched macrostructural and microstructural alterations in migraine, but few have focused on MOH patients and their results are conflicting. Concerning grey matter volume, some studies found significant modifications, mostly decreased volume [28, 35, 36], whereas other studies did not identify any significant changes [4, 5]. The same observation could be applied to white matter analyses, since some studies did not identify any significant alteration [36], whereas others, such as Chong & Schwedt’s one found increased diffusivity in thalamus, inferior longitudinal fasciculi and corticospinal tract, but no FA modification [37]. All these studies used standardized anatomical brain space analyses which require a normalisation step of MRI images. Standard space procedures have a higher probability of incorrectly labelling voxels due to the heterogeneity of cortical folds across subjects [38, 39]. The use of standard space procedures could be a source of serious bias, especially in disease which affects a varied population in term of both gender and age, such as migraine. Native-surface parcellation and surface-based registration may not be totally immune to the effects of anatomical variability, but they still have important advantages over methods based on transformation to a volume atlas. In particular, the accuracy of surface generation—the step that determines whether cortical grey matter is correctly identified—is easily evaluated by visual inspection on a slice-by-slice basis (a process described in more details in FreeSurfer documentation and in Seibert and Brewer [7]). A primary concern with volume–atlas analysis is that individuals’ cerebrospinal fluid and white matter may be interpreted as grey matter in atlas space (and vice versa) due to small, local registration errors that can be widespread and difficult to identify or correct. These errors are more readily avoided in the surface-based methods because tissue segmentation is performed, inspected, and, if necessary, manually corrected using the native images. It has already been demonstrated by direct comparison that the single step of registration to a volume atlas may affect the outcome of fMRI analysis and introduce large changes in resting fMRI correlations [7, 39].

## Limitations

A first limitation of this study issues from the number of statistical tests carried-out in the regional volumes without correction for multiple comparisons. The data discussed above must therefore be considered as exploratory, and needing confirmation with larger effectives. A second limitation concerns the hippocampal tractography, for which we were not able to distinguish fibers of afferent or efferent pathways. Identification of the origin of limbic alterations in MOH with different tractography techniques and separated ROIs would thus be worthwhile to delineate the role of the distinct pathways within the pathophysiology of the disorder.

## Conclusion

The use of semi-automated methods in native space appears to be adequate and more specific than using a common space for investigating brain morphometry in populations of different age and gender. This method permits to not focus the exploration on a priori regions of interest and is not as labor-intensive as drawing ROIs manually on all individual brain surfaces; it nevertheless offers a greater opportunity to detect group differences compared to the transformation of anatomical data into standard space. In the present study, the use of such methods has permitted to add evidence for a grey matter alteration which would characterize MOH pathophysiology in two manners: first being asymmetrical and second being regionally specific.

## Abbreviations

3D-MPRAGE: three-dimension magnetization-prepared rapid gradient-echo
ADC: apparent diffusion coefficient
BDI: Beck Depression Inventory
BIS: Barrat Impulsivity Scale
DMN: default mode network
EPI: echo planar imaging
FA: fractional anisotropy
FLAIR: fluid-attenuated inversion recovery
FOV: field of view
HC: healthy controls
ICHD-II: 2nd edition of international classification of headache disorders
IGT: Iowa Gambling Task
MDQ-H: Medication Dependence Questionnaire for Headache sufferers
MOH: medication overuse headache
MRI: magnetic resonance imaging
PCS: Pain Catastrophising Scale
ROI: region of interest
STAI: State Anxiety Inventory
TE: echo time
TI: inversion time
TR: repetition time
